# Psilocybin Enhances Cued Fear Extinction and Extinction Recall in Stress-Naïve, Acutely Stressed, and Chronically Stressed Mice

**DOI:** 10.1021/acsptsci.5c00462

**Published:** 2025-09-11

**Authors:** John Razidlo, Noelle Cataldo, Cody J Wenthur

**Affiliations:** † Neuroscience Training Program, School of Medicine and Public Health, University of Wisconsin, Madison, Wisconsin 53705, United States; ‡ Molecular and Cellular Pharmacology Training Program, School of Medicine and Public Health, University of Wisconsin, Madison, Wisconsin 53705, United States; § School of Pharmacy, University of Wisconsin, Madison, Wisconsin 53705, United States; ∥ Transdisciplinary Center for Research in Psychoactive Substances, University of Wisconsin, Madison, Wisconsin 53705, United States

**Keywords:** psilocybin fear conditioning, cortisol, stress, anxiety

## Abstract

Serotonergic psychedelics have shown promise in clinical trials for treating an array of mental health disorders, including depression, anxiety, and post-traumatic stress disorder. Despite these findings, our understanding of how these drugs mechanistically exert their therapeutic effects remains incomplete. While researchers have regularly employed rodent preclinical models to assess such mechanisms, many of these findings arise from stress-naïve animals. Given that prior environmental stress is a critical component for the mental health disorders being studied in clinical trials of psychedelics, understanding the performance of these drugs in animals previously exposed to acute or chronic stress is of strong translational relevance. In this study, we examined the effects of psilocybin in male mice that were stress-naïve, as well as in those that underwent either single-prolonged stress (SPS) or chronic restraint stress (CRS). The effects of these treatments on corticosterone release, extinction of freezing behavior, and recall of extinction in Pavlovian fear conditioning were examined for each group. We observed that psilocybin challenge transiently increased serum corticosterone in stress-naïve mice relative to saline; however, this effect was not observed in SPS and CRS animals. Interestingly, psilocybin treatment enhanced fear extinction and promoted extinction recall 24 h later not only in stress-naïve animals but also in stressed animals. These findings indicate psilocybin’s ability to acutely enhance fear extinction and promote enhanced extinction recall across animals with diverse environmental stress experiences prior to exposure.

## Graphical abstract

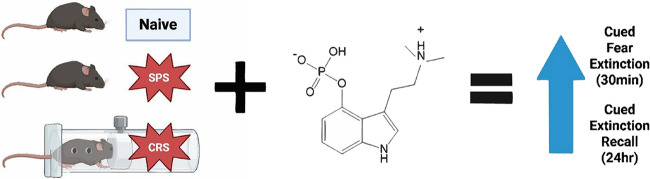

According to the Substance Abuse and Mental Health Services Administration (SAMHSA), over one-fifth of adults suffered from one or more mental health disorders, such as depression and anxiety, in the United States as of 2022.^1^ Not only are these mental health disorders accompanied by detriments in physical, mental, and social well-being, they are also a source of serious financial burden.^2–4^ Currently available treatment options, such as selective serotonin reuptake inhibitors (SSRIs), often fail to bring forth meaningful and durable improvements in mood and affect.^5,6^ This is evidenced by nearly 35, 50, and 33% of individuals being defined as treatment resistant for depression, anxiety, and post-traumatic stress disorder (PTSD), respectively.^6–10^ This has pushed researchers to either discover new and alternative treatment options, or to repurpose existing compounds for the purpose of treating mental health disorders. Regarding the latter approach, there has been a renewed interest in utilizing psychedelic compounds as interventions for a range of mental health disorders. Clinical trials employing various serotonergic psychedelics, such as psilocybin, LSD, and the monoaminergic entactogen MDMA, have shown promise in treating patients with mental health disorders including depression, anxiety, and PTSD.^11–16^ Despite these encouraging results, our understanding of the therapeutic mechanisms that drive these improvements in health and well-being, as well as the clinical and contextual factors that influence their interindividual efficacy, are incompletely understood.

Although the exact causes leading to the development of psychiatric disorders are not fully elucidated, there is a strong correlation between heightened and chronic stress and the development of psychiatric disorders.^17–20^ Chronic stress can lead to altered functioning of some of the body’s endogenous stress response systems, such as the Hypothalamic-Pituitary-Adrenal (HPA) Axis.^17,21^ This relationship is of particular interest given prior results demonstrating that psilocybin’s postacute and long-term reductions in anxiety-like behavior in mice were able to be blocked by prior HPA axis activation in the form of exogenous corticosterone administration.^22^ Furthermore, these studies demonstrated that the administration of the glucocorticoid receptor antagonist, mifepristone, prior to psilocybin administration was also able to block the stress-resilient phenotype otherwise observed. These results demonstrate that prior perturbation of the HPA axis, and particularly chronic HPA axis activation, can diminish the ability of psilocybin to induce lasting behavioral effects in mice. However, more information is needed to identify the specific conditions and contexts for which this is the case.

Because real-world HPA axis activation does not come from exogenously administered corticosteroids, the present work was designed to build off these prior results using two established environmental modes of stress-induction: single-prolonged stress (SPS) and chronic restraint stress.^23–27^ To assess whether HPA axis dysregulation was relevant for acute threat (“fear”) and potential threat (“anxiety”) responses, this work examined effects on fear learning and amelioration of stress-induced deficits through psilocybin administration. While there is evidence that emotional learning and stress coping are disrupted in patients suffering from psychiatric disorders,^28–30^ studying fear, anxiety, depression, emotional learning, and stress can be difficult from a translational perspective, as these are inherently human constructs. To help remedy this issue, researchers such as Fanselow and Hoffman have proposed studying the natural behavior of animal models and translational concepts such as threat assessment, which was proposed in their Predatory Immanence Continuum Theory (PICT).^31,32^ Additionally, in 2009, the National Institute of Mental Health (NIMH) introduced the Research Domain Criteria Initiative Framework (RDoC) for translational researchers studying mental health disorders, which highlights the use of fear conditioning experiments as a relevant paradigm to use when studying fear in preclinical animal models.^33^ Therefore, the ability of C57BL/6J mice to learn and extinguish negative stimulus-environment pairings using the associative learning task of fear conditioning was chosen for examination in this work.

To date, there are only six published articles exploring psilocybin-induced behavioral phenotypes in fear conditioning, one publication examining a psilocybin analog, 4-OH-DiPT, and one publication using *Psilocybe cubensis* extract; all of these conclude that drug administration reduces conditioned fear responses compared to their control counterparts, when such drug is provided shortly prior to extinction training.^34–41^ Interestingly, an ongoing, multisite study suggests that the behavioral effects of psilocybin, including its effects on fear extinction, are inconsistent across laboratories, suggesting the presence of additional, unexamined variables in influencing behavioral outcomes.^42^ Of the work examining psilocybin’s effects on fear conditioning, only one of these studies has examined the role of prior stress on psilocybin’s effects in this model, and all but one examined only the acute effects of psilocybin on fear conditioning. Further research examining the effects of stress on fear extinction and how it can be remedied is important, especially since prior stressors including SPS and CRS have been observed to impair fear extinction behavior.^25,43–46^ Given psilocybin’s ability to rapidly enhance plasticity in the acute phase, we hypothesize that animals that receive psilocybin will display a greater propensity to extinguish their previously acquired fear during fear extinction compared to their saline control counterparts. This paper provides the first measurements of the effects of psilocybin in prestressed SPS and CRS animals undergoing fear conditioning and extinction.

## Materials and Methods

All procedures were approved by the University of Wisconsin-Madison’s Institutional Animal Care and Use Committee (protocol #: M006074). Experiments were also conducted in full accordance with the NIH Guide for the Care and Use of Laboratory Animals.^47^ All drugs were handled by authorized users on the Schedule I DEA research license, and WI Special Use Authorizations held by Dr. Cody Wenthur. See Supporting Information and Methods for details on psilocybin sources, preparation, and administration.

### Psilocybin

Psilocybin was handled by authorized users on the Schedule I DEA research license and WI Special Use Authorizations held by Dr. Cody Wenthur. Psilocybin powder (Usona Institute; Madison, WI) was prepared immediately prior to use and administered via IP injection as previously described.^22^ Psilocybin doses were chosen based off of previous behavioral effects on threat-response behaviors that were observed in our laboratory, along with evidence from other fear conditioning studies that employ the use of psilocybin or related analogs.^22,35,36,38,40^

### Animals

All mice used in this work were acclimated to University of Wisconsin vivarium conditions for at least seven-days prior to handling or experimentation. All C57BL/6J mice (7–9 weeks old males; The Jackson Laboratory, Maryland, USA) were housed in pairs, while under a 12 h artificial reverse light/dark cycle. Room temperature remained constant between 22–24 °C. All experiments took place during the animals’ dark cycle. All experimental procedures were completed in full accordance with Research Animal Resources and Compliance (RARC) guidelines in Association for Assessment and Accreditation of Laboratory Animal Care (AAALAC) accredited facilities. Mice were the selected animal model of choice as previous published studies have used this species to examine the effects of psilocybin on fear conditioning.^35,36,38^ Furthermore, previous work published by our group has also studied the effects of psilocybin in C57BL/6J mice.^22^ Only male animals were used in this study, as previous work has suggested that male mice display greater cue-induced freezing compared to female mice,^48^ whereas other rodent studies have observed increases in darting behavior in females compared to males.^49^

### Fear Conditioning

Fear conditioning chambers and their paired software were purchased from Maze Engineers (Skokie, IL, USA). In all experiments, the unconditioned stimulus (US) used was a mild foot-shock (0.4 mA, 2 s) and the conditioned stimulus (CS) used was a 30 s (1000 Hz) auditory tone. During CS-US pairings, foot shock coterminated with the auditory tone. All acquisition experiments (context A) began with a 4 min stimulus-free period, followed by 8 CS-US stimulus presentations separated by 30 s, and ending with another 1 min stimulus-free period. Both extinction and recall sessions (context B) began with a 4 min stimulus-free period, followed by 12 stimulus presentations (CS only) separated by 30 s, and ending with another 1 min stimulus-free period. Behavioral sessions were separated by 24 h (Figure 1A). Behavioral chambers were thoroughly cleaned with a 70% ethanol solution when switching out animals.

**Figure 1:**
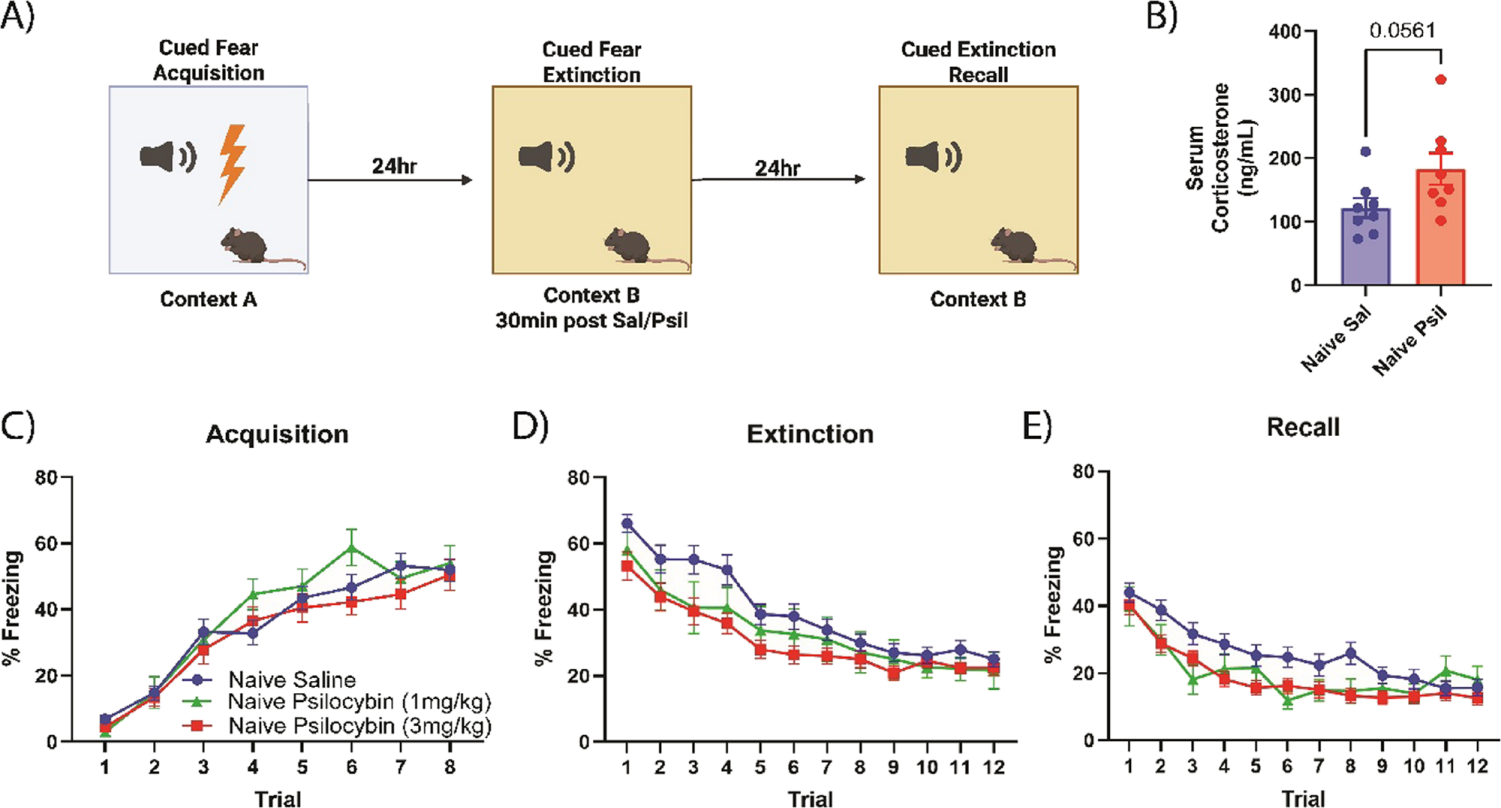
Psilocybin enhances fear extinction and recall in stress-naïve mice. (A) Depiction of 3-day fear conditioning and extinction schedule. (B) Posttreatment (15 min) serum corticosterone levels in a separate cohort of animals (Unpaired T-test with Welch’s correction, *F*_(7,7)_ = 2.591, *p* = 0.0561). Psilocybin challenge at 3 mg/kg modestly increased serum corticosterone compared to saline control. (C) Fear acquisition freezing time course. (D) Time series of freezing during cued fear extinction (two-way ANOVA, time *x* treatment interaction, *F*_(11, 539)_ = 2.738, *p* = 0.0019; main effect of treatment, *F*_(5.063, 248.1)_ = 66.73, *p* = 0.0152). Animals that received psilocybin showed reduced freezing during the extinction session, with 3 mg/kg psilocybin showing the greatest reductions in freezing. (E) Time series of freezing during fear extinction recall (two-way ANOVA, time x treatment interaction, *F*_(22,638)_ = 2.747, *p* < 0.0001). Behavioral data: saline *n* = 30, psilocybin 3 mg/kg *n* = 24, psilocybin 1 mg/kg *n* = 10; corticosterone data: naïve saline *n* = 8, naïve psilocybin *n* = 8; data represented as the mean ± SEM.

### Contextual Environments

Contextual environments consisted of different visual, odor, auditory, and tactile cues. During these experiments, two separate contextual environments were used.

#### Contextual Environment 1

As a visual cue, this square-shaped chamber was lit by a blue light, and the borders of this chamber were covered in blue cloth. The odor cue used in this environment was 1 mL of 10% vanilla extract placed in a dish below the flooring of the chamber. A low-powered fan was turned on to provide white-noise as an auditory contextual cue in this chamber. When used as the acquisition contextual environment, the flooring consisted of stainless-steel metal bars. When used as the extinction or recall behavioral chamber, a plastic mesh was placed over the metal bar flooring to provide a different tactile cue.

#### Contextual Environment 2

As a visual cue, this square-shaped chamber was lit by a white light. The odor cue used in this environment was 1 mL of 10% peppermint extract placed in a dish below the flooring of the chamber. To further differentiate this environment from that of contextual environment 1, no low-powered fan was used. When used as the acquisition contextual environment, the flooring consisted of stainless-steel metal bars. When used as the extinction or recall behavioral chamber, a plastic mesh was placed over the metal bar flooring to provide a different tactile cue.

### Behavioral Freezing

Behavioral freezing was scored automatically using Any-maze (Wood Dale, IL, USA). Behavioral freezing in this paper is defined as the complete absence of movement except for movement due to respiration. Using the default freeze-detection sensitivity settings, animals needed to remain frozen for 1 s before they would be scored as freezing by the software. Freezing values displayed in this paper are the percent freezing during each 1 min trial. Additionally, any animals that did not display greater than 5% freezing in at least one trial during the fear acquisition training session were omitted from the study (*n* = 3).

### Single-Prolonged Stress (SPS) Induction

Animals undergoing SPS were placed in 50 mL conical falcon tubes for 2 h. Immediately upon removal from restraint stress, animals were placed into a container of water (28 °C ± 1°, 8 in. in depth) for 20 min. Upon completion of the forced swim, animals were dried off and then underwent isoflurane anesthesia for 2 min. Subsequently, animals were placed back into their home cage and monitored until they recovered from anesthesia. Animals were given a week to recover before further testing. Notably, our protocol utilized isoflurane anesthesia per our approved IACUC protocol rather than diethyl ether.^24,26,50^

### Chronic Restraint Stress (CRS) Induction

Animals undergoing CRS were placed in 50 mL conical Falcon tubes for 1 h per day, every day, for 1 week. Restraint stress began at the same time each day. Upon removal from the tubes, animals were returned to their home cage. After the final restraint stress session, animals were given 24 h to recover in their home cage before further testing.

### Corticosterone

Endogenous corticosterone levels were measured via corticosterone enzyme-linked immunosorbent assays (ELISAs) purchased from Enzo Biochem Inc. (Farmingdale, NY). Serum corticosterone samples were collected with separate cohorts of animals from those that underwent behavioral analyses to avoid behavioral alterations due to the sample collection process. Animals were anesthetized via isoflurane in a drop jar briefly for blood collection via retro-orbital (RO) bleeds. Blood collection for corticosterone ELISA experiments occurred immediately prior to treatment administration to assess baseline values, and 15 min after treatment administration. SPS corticosterone measurements were collected immediately following stress exposure and at 6 days after stress exposure. CRS corticosterone measurements were collected on day 8 after the initial stress exposure. Samples were collected and immediately placed on ice, then centrifuged at 10,000 rpm for 10 min. The resulting serum fraction was collected and stored at −80 °C until just before undergoing ELISA testing.

### Statistical Analysis and Data Presentation

Statistical analyses were performed using GraphPad Prism, version 11 (San Diego, CA, USA). All tests were run as two-tailed analyses, setting *p* < 0.05 as the threshold for significance. Data analyzed across time were assessed using repeated measures approaches; all samples were otherwise considered to be independent for analysis purposes. One and two-way ANOVA analyses, as well as their nonparametric analogues, were corrected for multiple comparisons when assessing differences across each condition using Šídák’s multiple comparisons test. Where indicated, *n*, number of animals. All data are shown as mean ± SEM.

## Results

### Psilocybin Enhances Fear Extinction and Recall in Stress-Naïve Animals

Following fear conditioning on day 1 (Figure 1A), animals were pseudorandomly assigned to treatment groups to ensure that there were no baseline differences in the initial fear acquisition phase. All three groups of animals showed a robust acquisition of fear memory (Figure 1C; two-way ANOVA, main effect of time, *F*_(5.009, 290.5)_ = 124.7, *p* < 0.0001). On day 2, animals underwent cued fear extinction in a different contextual environment, and received their assigned drug treatment (saline, 1 mg/kg psilocybin, 3 mg/kg psilocybin) via intraperitoneal (IP) injection, 30 min prior to fear extinction training (Figure 1D). All groups displayed marked reductions in freezing as the extinction trials progressed (two-way ANOVA, main effect of time, *F*_(5.628, 326.4)_ = 63.45, *p* < 0.0001). Psilocybin-treated (3 mg/kg) animals displayed enhanced fear extinction compared to their saline-treated counterparts (two-way ANOVA, time *x* treatment interaction, *F*_(11, 539)_ = 2.738, *p* = 0.0019; main effect of treatment, *F*_(5.063, 248.1)_ = 66.73, *p* = 0.0152). Although animals treated with 1 mg/kg psilocybin displayed some reductions in absolute freezing values, this lower dose did not significantly reduce freezing compared to saline controls in the paradigm used here (two-way ANOVA, time x treatment interaction, *F*_(11, 396)_ = 1.017, *p* = 0.4301; main effect of treatment, *F*_(1,36)_ = 1.277, *p* = 0.2659). On day 3 both groups were reintroduced to the same contextual environment used in fear extinction and underwent a fear extinction recall test to assess the retention of their extinction memory (Figure 1E). Despite receiving psilocybin treatment more than 24 h prior to the fear recall test, animals that had received either dose of psilocybin prior to extinction displayed reduced freezing during recall compared to their saline counterparts, with a main effect of treatment (two-way ANOVA, time *x* treatment interaction, *F*_(22,638)_ = 2.747, *p* < 0.0001). Interestingly, animals that received 3 mg/kg psilocybin displayed significantly less freezing compared to animals that received 1 mg/kg psilocybin prior to extinction (two-way ANOVA, time x dose interaction, *F*_(11,341)_ = 1.921, *p* = 0.0359). These results demonstrate that psilocybin treatment leads to enhancements in fear extinction and/or recall in stress-naïve mice at doses of up to 3 mg/kg. Based off these results, all experiments examining prior stress exposure used psilocybin at a dose of 3 mg/kg.

Acute psilocybin administration has been reliably demonstrated to lead to a transient increases in serum corticosterone for rodents and humans.^22,51–53^ In mice, this HPA axis response appears to be necessary for the postacute behavioral effects of psilocybin, and can be blocked by blunting this effect with either acute or chronic administration of exogenous corticosterone or through the use of mifepristone.^22^ To assess the acute effects of psilocybin on HPA axis functioning in our model, animals were treated with either IP saline or 3 mg/kg psilocybin and underwent serum collection 15 min after treatment administration (Figure 1B). Animals that received psilocybin displayed modest increases (Unpaired T-test with Welch’s correction, *F*_(7,7)_ = 2.591, *p* = 0.0561) in serum corticosterone compared to their saline counterparts. These findings, paired with the behavioral results, suggest that this transient increase in serum corticosterone after psilocybin administration may also be important for psilocybin’s acute behavioral effects in Pavlovian fear conditioning and extinction.

### Psilocybin Rescues Stress-Induced Deficits in Pavlovian Fear Conditioning in Animals Exposed to a Single, Temporally Distant Stressor

In addition to assessing the effects of psilocybin in stress-naïve animals, the effects of a single, intense event of stress in the form of single-prolonged stress (SPS) on Pavlovian fear conditioning and extinction were also examined (Figure 2A). On day one, C57BL/6J males, underwent SPS. After SPS, animals were returned to their home cage for 6 days. First, we set out to assess the effects of SPS on HPA axis activation, as measured by serum corticosterone, and how this mode of stress may modulate the effects of psilocybin. Compared to handled controls, animals that had just completed SPS had greatly increased serum corticosterone (Unpaired T-test with Welch’s correction, *F*_(11,11)_ = 5.270, *p* < 0.0001) (Figure 2B). This increase in serum corticosterone was no longer observed 6 days later (Unpaired T-test with Welch’s correction, *F*_(7,7)_ = 2.990, *p* = 0.1243) (Figure 2C). When challenged by drug treatment, there was no observed increase in serum corticosterone in SPS animals treated with psilocybin compared to their saline counterparts (Unpaired T-test with Welch’s correction, *F*_(3,3)_ = 2.273, *p* = 0.3824), with psilocybin-treated animals even displaying slightly lower levels of serum corticosterone (Figure 2D). These results suggest that prior stress in the form of SPS reduces HPA axis responses to subsequent psilocybin challenge for up to 1 week later.

**Figure 2:**
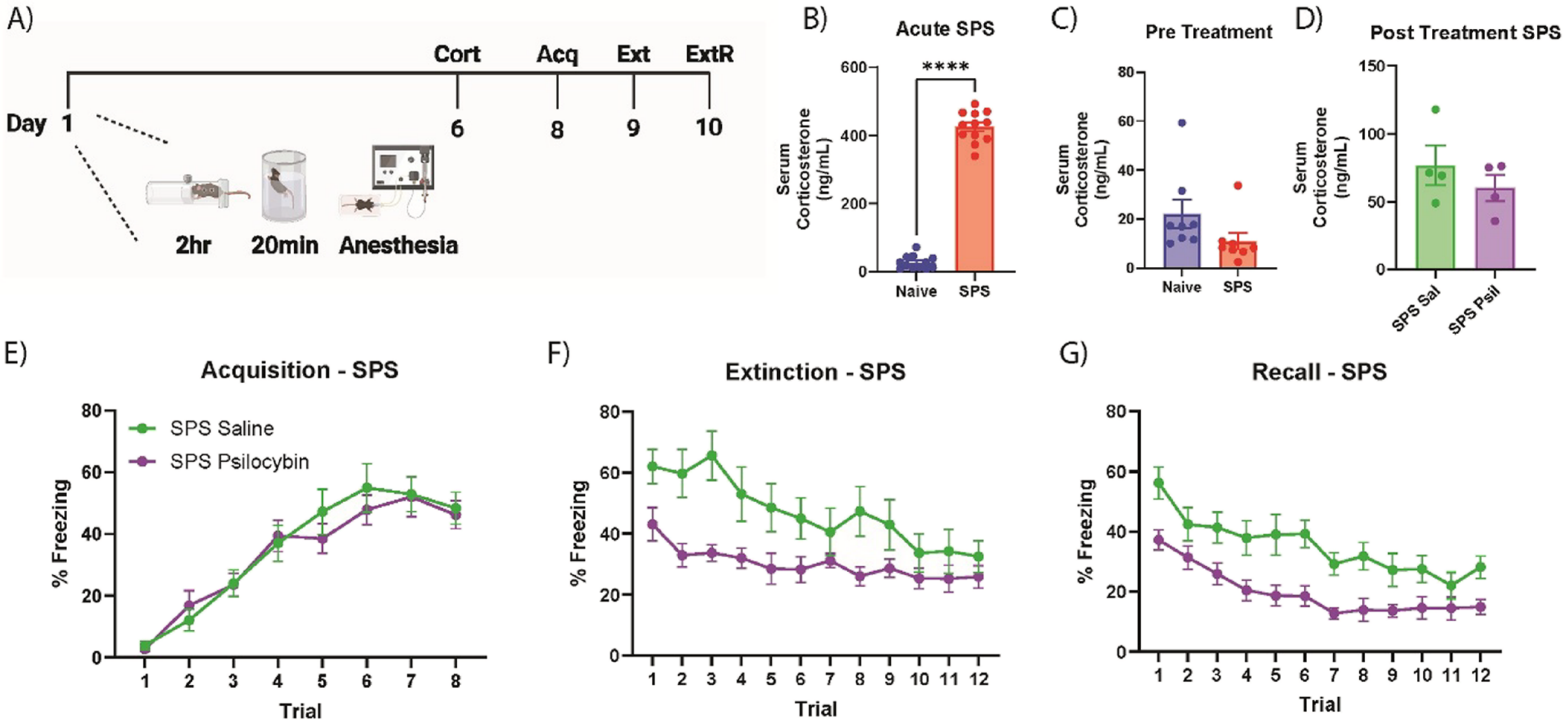
Psilocybin rescues deficits in fear extinction induced by SPS. (A) Depiction of experimental timeline. Animals underwent single-prolonged stress (SPS) on day 1, which consists of 2 h restraint stress, immediately followed by 20 min forced swim stress, immediately followed by anesthesia. (B) Acute effects of SPS on serum corticosterone in a separate cohort of animals (unpaired T-test with Welch’s correction, *F*_(11,11)_ = 5.270, *p* < 0.0001). Acutely, SPS greatly increased serum corticosterone compared to handled controls. (C) Pretreatment serum corticosterone levels. (D) Posttreatment (15 min) serum corticosterone in SPS animals treated with either saline or psilocybin. (E) Time series of freezing during fear acquisition training. (F) SPS animals that received single injection of psilocybin (3 mg/kg), 30 min prior to fear extinction training displayed enhanced extinction compared to saline counterparts (two-way ANOVA, time x treatment, *F*_(11, 198)_ = 2.869, *p* = 0.0016; main effect of treatment, *F*_(1, 18)_ = 5.832, *p* = 0.0266). G) SPS animals that received psilocybin treatment prior to extinction displayed reduced freezing during the extinction recall test compared to their saline treated counterparts (two-way ANOVA, main effect of treatment, *F*_(1, 18)_ = 11.99, *p* = 0.0028). Behavioral data: *n* = 12 per group; corticosterone data: naïve *n* = 8, SPS *n* = 8, SPS saline *n* = 4, SPS psilocybin *n* = 4; data represented as the mean ± SEM. SPS, single-prolonged stress; Acq, fear acquisition; Ext, fear extinction; ExtR, extinction recall test.

After the week-long SPS recovery period, both groups underwent cued fear conditioning (Figure 2E). Behavioral freezing was assessed, and animals were pseudorandomly assigned to drug treatment groups to ensure that there were no differences in behavioral freezing between groups arising from performance differences during the fear acquisition phase (two-way ANOVA, time x treatment interaction, *F*_(7,126)_ = 0.7501, *p* = 0.6300). Both groups showed a robust effect of time (two-way ANOVA, main effect of time, *F*_(4.299, 77.39)_ = 50.48, *p* < 0.0001), indicating both groups acquired a fear memory. The next day, animals underwent cued fear extinction in a different contextual environment and received their assigned drug treatment via IP injection 30 min prior to fear extinction training (Figure 2F). Both groups displayed marked reductions in freezing as the extinction trials progressed (two-way ANOVA, main effect of time, *F*_(4.919, 88.55)_ = 11.21, *p* < 0.0001). Of note, saline animals that underwent SPS displayed impaired fear extinction compared to their stress naïve counterparts (two-way ANOVA, time *x* stressor, *F*_(11, 396)_ = 2.013, *p* = 0.0260), demonstrating the efficacy of SPS in altering fear extinction learning. Similar to the results seen in the stress-naïve animals, SPS animals that received IP psilocybin (3 mg/kg) also displayed enhanced fear extinction compared to their saline-treated counterparts (two-way ANOVA, time *x* treatment, *F*_(11, 198)_ = 2.869, *p* = 0.0016; main effect of treatment, *F*_(1, 18)_ = 5.832, *p* = 0.0266). Strikingly, psilocybin-treated SPS animals displayed enhanced extinction compared to saline-treated naïve animals (two-way ANOVA, time *x* group, *F*_(5.617,202.2)_ = 6.827, *p* < 0.0001). One day later, both groups were reintroduced to the same contextual environment used in fear extinction and underwent a fear extinction recall test to assess the retention of their extinction memory (Figure 2G). Again, despite receiving psilocybin treatment more than 24 h prior to the fear recall test, SPS animals that received psilocybin prior to extinction displayed reduced freezing compared to their saline counterparts (two-way ANOVA, main effect of treatment, *F*_(1, 18)_ = 11.99, *p* = 0.0028). Additionally, saline-treated animals that underwent SPS showed moderately elevated freezing during the recall test compared to their naïve counterparts (two-way ANOVA, main effect of stressor, *F*_(1, 36)_ = 3.717, *p* = 0.0618), whereas there were no such differences in fear recall testing between psilocybin-treated animals across the naïve and SPS groups (two-way ANOVA, time *x* stressor interaction, *F*_(11,341)_ = 0.4667, *p* = 0.9231; main effect of stressor, *F*_(1,31)_ = 0.1062, *p* = 0.7467). Paired with the corticosterone ELISA results, these results suggest that psilocybin ameliorates SPS-induced fear extinction impairment during the acute phase, even in animals that have impaired HPA axis responses to psilocybin challenge, and these improvements are carried over well beyond drug clearance from the system, as evidenced by the reduced freezing during the extinction recall testing the following day.

### Psilocybin Rescues Stress-Induced Deficits in Pavlovian Fear Conditioning in Animals Exposed to a Chronically Repeated Stressor

In addition to examining the effects of psilocybin in stress naïve animals and animals that underwent a single, intense episode of stress, we also sought to assess the effects of psilocybin in animals that underwent repeated stress (Figure 3A). In this paradigm, C57BL/6J males underwent seven consecutive days of restraint stress for 1 h each day. Animals that underwent CRS showed higher levels of serum corticosterone compared to stress-naïve handled controls immediately prior to treatment administration (Unpaired Ttest with Welch’s correction, *F*_(6,7)_ = 12.88, *p* = 0.0126) (Figure 3B). Furthermore, animals that underwent CRS that received a subsequent psilocybin challenge did not show any elevations in corticosterone compared to their saline-treated counterparts (Unpaired Ttest with Welch’s correction, *F*_(3,2)_ = 3.664, *p* = 0.9389) (Figure 3C), indicating reduced HPA axis response to subsequent psilocybin challenge, akin to that observed for SPS experiments.

**Figure 3:**
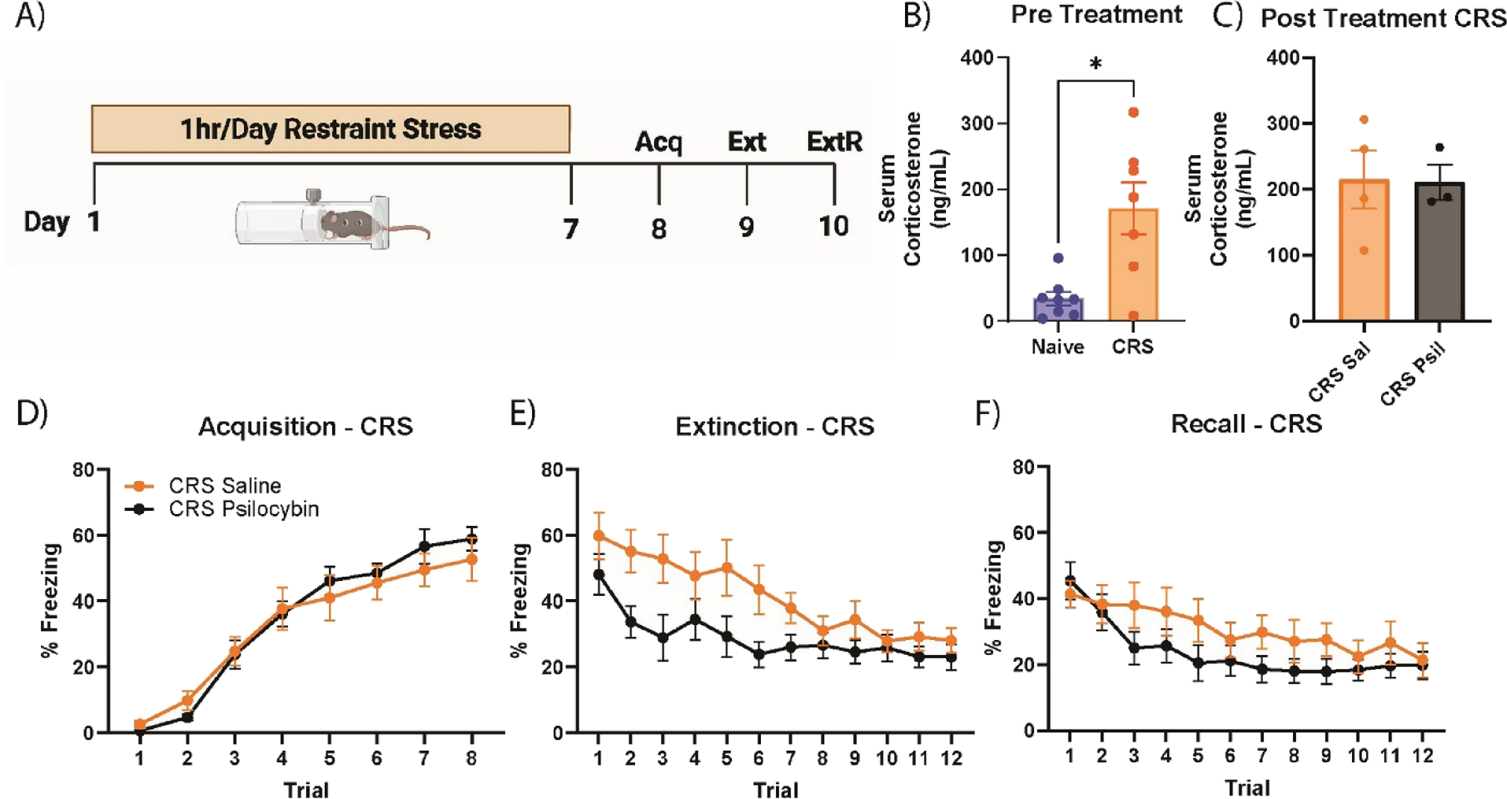
Psilocybin rescues deficits in fear extinction induced by CRS. (A) Depiction of experimental timeline. (B) Pretreatment serum corticosterone levels between a separate cohort of naïve and CRS animals from behavioral testing (unpaired T-test with Welch’s correction, *F*_(6,7)_ = 12.88, *p* = 0.0126). Animals that underwent prior CRS stress had elevated levels of corticosterone compared to handled controls. (C) Posttreatment (15 min) serum corticosterone levels between saline and psilocybin-treated CRS animals. (D) Time series of freezing during fear acquisition training. Animals were assigned to drug treatment groups after fear acquisition. (E) CRS animals that received single injection of psilocybin (3 mg/kg), 30 min prior to fear extinction training displayed enhanced extinction compared to saline counterparts (two-way ANOVA, time *x* treatment interaction, *F*_(11,242)_ = 2.737, *p* = 0.0024). (F) CRS animals that received psilocybin treatment prior to extinction displayed reduced freezing during the extinction recall test compared to their saline treated counterparts (two-way ANOVA, time *x* treatment interaction, *F*_(5.803,127.7)_ = 1.865, *p* = 0.0942). Behavioral data: *n* = 12 per group; corticosterone data: naïve *n* = 8, CRS *n* = 7, CRS saline *n* = 4, CRS psilocybin *n* = 3; data represented as mean ± SEM. CRS, chronic restraint stress; Acq, fear acquisition; Ext, fear extinction; ExtR, extinction recall test.

One day after the final restraint stress, animals went through Pavlovian fear conditioning with behavioral freezing assessed (Figure 3D). Once again, animals were pseudorandomly assigned to drug treatment groups to ensure that there were no differences in behavioral freezing between them during the fear acquisition phase (two-way ANOVA, time x treatment interaction, *F*_(7,154)_ = 0.8627, *p* = 0.5375). Both groups showed a robust effect of time (two-way ANOVA, main effect of time, *F*_(4.189,92.15)_ = 72.89, *p* < 0.0001), indicating both groups acquired a fear memory. The next day, animals underwent cued fear conditioning in a different contextual environment, and received their assigned drug treatment via IP injection, 30 min prior to fear extinction training (Figure 3E). Both groups displayed marked reductions in freezing as the extinction trials progressed (two-way ANOVA, time *x* treatment interaction, *F*_(4.634,102.0)_ = 15.23, *p* < 0.0001).

Saline animals that underwent CRS displayed modest impairments in fear extinction compared to their stress naïve counterparts (two-way ANOVA, time *x* treatment interaction, *F*_(11,418)_ = 1.696, *p* = 0.0717). Chronically restrained animals that received IP psilocybin (3 mg/kg) displayed enhanced fear extinction compared to their saline-treated counterparts (two-way ANOVA, time x treatment interaction, *F*_(11,242)_ = 2.737, *p* = 0.0024). Interestingly, as was seen with SPS animals, psilocybin-treated CRS animals displayed enhanced extinction compared to naïve saline animals (two-way ANOVA, time *x* group, *F*_(5.429,206.3)_ = 5.723, *p* < 0.0001; main effect of group, *F*_(1,38)_ = 4.798, *p* = 0.0347). One day later, both groups were reintroduced to the same contextual environment used in fear extinction and underwent a fear extinction recall test to assess the retention of their extinction memory (Figure 3F). Despite receiving the treatment more than 24 h prior to the fear recall test, chronically restrained animals that received psilocybin displayed modestly reduced freezing compared to their saline counterparts during the recall test session (two-way ANOVA, time *x* treatment interaction, *F*_(5.803,127.7)_ = 1.865, *p* = 0.0942). Of note, saline-treated animals that underwent CRS showed elevated freezing during the recall test compared to their naïve counterparts (two-way ANOVA, time *x* stressor interaction, *F*_(11,418)_ = 1.961, *p* = 0.0308). Interestingly, there were no observed differences in freezing behavior between the psilocybin-treated naïve and CRS animals (two-way ANOVA, time *x* stressor interaction, *F*_(11,363)_ = 0.4125, *p* = 0.9504; main effect of stressor, *F*_(1,33)_ = 0.1920, *p* = 0.1752). Similar to what was observed in SPS animals, these results suggest that psilocybin can enhance fear extinction during the acute phase, even in animals that have undergone repeated restraint stress and have impaired HPA axis responsivity to psilocybin challenge; interestingly, psilocybin’s effects on recall of an extinguished fear memory may be somewhat weaker when preceded by chronic versus acute stressors.

## Discussion

Here, we observed that a high dose of psilocybin enhances fear extinction after a single dose in stress-naïve animals, with psilocybin-treated animals also showing reduced freezing during recall testing the following day at doses up to 3 mg/kg. Given that these effects are observed well beyond the time-course of drug exposure, this indicates that decreases in freezing are not arising exclusively due to perceptual or motoric disturbances. These results comport with what other research groups have observed regarding psilocybin and its analogs during fear conditioning and extinction.^35,36,38–41,54^ Furthermore, congruent with previous studies in our lab, we observed an increase in serum corticosterone shortly after psilocybin administration in naïve animals.^22^ An ongoing study also has also observed increases in serum corticosterone after psilocybin administration, attributed in part by CRH neuron activation, which is reporting similar results to what we have observed in this work.^55^ As expected, prior HPA axis perturbation, now in the form of SPS and CRS, was also observed to block psilocybin-induced increases in serum corticosterone. Surprisingly, however, this blunting of psilocybin-induced corticosterone secretion did not block psilocybin’s behavioral effects in SPS and CRS stressed animals in Pavlovian fear conditioning and extinction. That is, despite reduced HPA axis responsivity to psilocybin challenge in both SPS and CRS animals, psilocybin treatment enhanced fear extinction regardless of prior stress, with evidence of reduced freezing during recall testing as well. This observation of maintenance of psilocybin’s effects in reduction of acute-threat (fear) associated behaviors despite HPA axis downregulation stands in contrast with prior observations of HPA axis downregulation leading to ablation of psilocybin-induced behavioral phenotypes regarding potential threat (anxiety) behaviors.^22^ There are several notable differences between the previous results and the results we present from this study that are likely to contribute to this difference including the animal model utilized, type of behavior measured, and phase of psilocybin exposure during this assessment.

First, as noted before, our previous study used exogenous corticosterone exposure as the main form of HPA axis intervention, whereas here we used SPS and CRS behavioral stressors.^22^ Corticosterone, although involved in stress response signaling, is only one component of the stress and stress-resolution response, often being oversimplified as a true stressor.^56^ Animals undergoing SPS and CRS stress, as employed here, are likely to recruit and activate a more complex set of endogenous stress-response processes than in the case of exogenous corticosteroid administration. Regardless, we did observe negative feedback on the HPA axis in both studies, evidenced by reduced corticosterone secretion with subsequent stressors.

The second notable difference is that previous behavioral assessments utilized the open-field (OF) and novelty suppressed feeding (NSF) tasks which are measures of approach and avoidance, commonly evaluated as representing anxiety-like behavior. These behavioral tasks require the animals to passively assess the environment for potential dangers. This contrasts with Pavlovian fear conditioning and extinction (FCE), which tasks the animals with actively learning and attending to cues that are predictive of an immediate threat. Although there is an overlap in the circuitry between anxiety and fear, there are key differences.^57,58^ Cued fear conditioning is heavily dependent on the amygdala, with amygdala lesions completely ablating fear responding, which was not the case in experiments where other limbic regions such as the hippocampus and medial prefrontal cortex were lesioned.^59–61^ Although involved in OF and NSF tasks, the contribution of the amygdala does not appear to be as great, with these behaviors recruiting more from other regions such as the bed nucleus of the stria terminalis (BNST).^61,62^ To get a more complete picture of the interplay between prior stress and psilocybin behavioral effects, a wider array of behavioral tasks, that engage a variety of central nervous system circuits, needs to be employed. Intriguingly, decreased activation of CRH-responsive neurons in the paraventricular nucleus of the hypothalamus in a novel, OF environment has now been observed following psilocybin administration.^55^ This activity reduction was suggested to result in a reduced ability of mice to interpret and respond to potential threats. Given the acute and lasting effects of psilocybin observed here, exploration of this phenomenon in the context of immediate threat and extinction of conditioned fear may provide additional insight into shared mechanisms across threat contexts.

The third, and perhaps most important difference, between this study and Jones et al. is that the behavioral results here were observed during the acute phase of psilocybin exposure, not after psilocybin’s clearance. Previous electrophysiological studies examining the role of serotonin receptor activation in the amygdala have found serotonin-induced inhibition of pyramidal cell firing, which appears to be dependent on GABA receptor activation.^63–65^ Interestingly, only recently have researchers examined this relationship in the presence of psychedelics, yielding evidence that psychedelic 5-HT_2A_ receptor agonists can inhibit amygdala output through direct agonism of 5-HT_2A_ receptors on GABA interneurons.^35^ This suggests that acute 5-HT_2A_ receptor agonism may be acting as the driving force for enhanced fear extinction in this context, largely occluding any modulatory effects arising from HPA axis activation and corticosterone activity. The relief of this occlusion following psilocybin clearance may help account for the more significant impact of HPA axis and glucocorticoid effects in the previously reported anxiety results.

Notably, as with our previous paper describing psilocybin-induced alteration of anxiety-like effects, this paper also only used male animals, as previous research suggests that male animals have greater cue-induced freezing responses.^48^ Future experiments should examine the interplay of psilocybin and stress in female animals, given there is evidence of sex differences in response to psilocybin and psilocybin analogs in other behavioral paradigms.^35,36,53^

Given the nature of the experiments presented in this work, it is worth contextualizing the findings of this study to the broader body of literature examining the effects of psychedelics on humans. From a translational perspective, the most relevant human behavioral task and intervention to extrapolate the findings from the cued fear conditioning used in this work is that of exposure therapy used in treating PTSD patients. Exposure therapy, although effective, does not come without limitations.^66^ In exposure therapy, already vulnerable participants must confront their most traumatic experiences, as well as triggering stimuli. Notably, there are high attrition rates observed in cases where exposure therapy is used. One meta-analysis examining dropout rates in various PTSD treatment regimens found that the average dropout rate was 18%.^67^ Strikingly, the dropout rate was double that at 36% when trauma-specific treatments such as exposure therapy were used. Furthermore, comorbidities such as substance use disorder have been correlated with even greater attrition rates, with one study finding that comorbid substance use disorder with PTSD led to attrition rates as high as 62%.^68^ Psilocybin’s enhancement of extinction, as well as enhanced recall of extinction, in mice previously exposed to a single-prolonged stress, suggest that approaches combining psilocybin with exposure therapy could be of potential therapeutic value. Critically, however, all completed preclinical studies to date, including this one, have demonstrated effects arising only following the presence of acute psychedelic exposure during extinction training. Whether the subjective state induced by ongoing psychedelic action in conjunction with active exposure therapy would actually enhance patient tolerability or reduce dropout in humans, rather than reducing tolerability and increasing dropout, is unexamined, and the design of such a study would demand extremely careful ethical consideration, given the vulnerable psychological state induced by psychedelic administration.

In summary, we demonstrated that psilocybin increases serum corticosterone and enhances fear extinction in stress-naïve mice. Next, we demonstrated that both single-prolonged stress and chronic restraint stress impaired future HPA axis responsivity to psilocybin challenge. Despite this, psilocybin was able to enhance fear extinction in animals regardless of prior stress exposure, which was also carried over to the following day during fear extinction recall testing. These are the first findings exploring psilocybin’s ability to enhance fear extinction in rodents across multiple stressors. Future studies should examine this relationship with other behaviors and across sexes.

## Abbreviations

AAALAC: Association for Assessment and Accreditation of Laboratory Animal Care
Acq: cued fear acquisition
BNST: bed nucleus of the stria terminalis
CRS: chronic restraint stress
CS: conditioned stimulus
DEA: Drug Enforcement Agency
ELISA: enzyme-linked immunosorbent assay
Ext: cued fear extinction
ExtR: cued extinction recall
FCE: cued Pavlovian fear conditioning
GABA: gamma aminobutyric acid
HPA: hypothalamic-pituitary-adrenal axis
IACUC: Institutional Animal Care and Use Committee
IP: intraperitoneal
LSD: lysergic acid diethylamide
MDMA: 3,4-methylenedioxymethamphetamine
NIMH: National Institute of Mental Health
NSF: novelty suppressed feeding test
OF: open field test
PICT: predatory imminence continuum theory
Psil: psilocybin
PTSD: post-traumatic stress disorder
RARC: Research Animal Care and Compliance
RDoC: research domain criteria initiative
RO: retro-orbital
Sal: saline
SAMSHA: Substance Abuse and Mental Health Services Association
SPS: single-prolonged stress
SSRI: selective serotonin reuptake inhibitor
US: unconditioned stimulus
4-OH-DiPT: 4-hydroxy-N,N-diisopropyltryptamine
5-HT_2A_: serotonin 2A receptor
